# A New Aluminum Process

**Published:** 1888-12

**Authors:** 


ARTICLE VI.

### A NEW ALUMINUM PROCESS.

Messrs. Brin Brothers, the inventors of the industrial process of separating the oxygen from the nitrogen of the atmosphere, recently showed some experiments in connection with a new process of making aluminum alloys, at their laboratory, 9 College Street, Belvedere Road, London. An ordinary but rich clay was mixed with a reducing agent called by them "a flux," and made into a paste with water. Some pig iron which had been run into bars three-eighths inch thick and two inches broad was broken into pieces. These pieces were charged with the paste and alternate layers of coke into a small cupola. A further quantity of coke to fill the furnace was put upon the top of the charge, and the blast from a fan turned on. In about twenty-five minutes the pig iron had melted. According to the inventors, nascent aluminum is produced in contact with the molten iron, and penetrates the same, the effect of the conbination being to reduce the melting point of both metals and to yield a more fluid product than either of them separately. The contents of the furnace were then discharged into a ladle, and castings were made of the "aluminum steel," containing about 1.75 per cent. of aluminum. The nature of the flux was not revealed, as Messrs. Brin have not yet completed all their patents, but the inventors state that its cost is not higher than that of the clay used. The castings were exceedingly sonorous, for when suspended by a string and struck with a piece of metal the vibrations lasted from thirty to forty-five seconds. The castings were of white fracture, and free from blow holes. The silicon and some other impurities of cast-iron are thrown out in the form of slag. The aluminum has thus a twofold function in this process. It forms definite alloys with the iron, and aids in clearing out its impurities.

In another experiment the ready manner in which aluminum can be reduced by the process was illustrated. A piece of thin, soft scrap iron was coated with the clay and flux, and inserted in a blow-pipe flame. At a bright yellow heat the clay was reduced, and metallic aluminum became occluded in the whole thickness of the iron, giving the latter a white surface. The resulting metal, instead of being soft and pliable, became tough and springy, and it was claimed had acquired all the properties of first-class steel. Some of the alloy thus made was put into strong, pure nitric acid, and was not acted upon thereby; while a piece of the original scrap iron was rapidly attacked under the same circumstances. The proportion of aluminum in the steel produced depends, within certain limits, upon the proportions employed of the original ingredients for charging the furnace. Alloys of copper and of some other metals can be formed in the same way. Some copper aluminum bronze was exhibited; also such a bronze alloyed with from 17 to 20 per cent. of steel. This alloy can be made hard, and with a fracture like fine cast steel; or by careful annealing and repeated rolling a fibrous texture can be produced. Mr. Frederick Varley, who has made experiments with Messrs. Brin's aluminum steel, states that it has all the properties of the best iron for conducting magnetism, while chilled castings will make excellent permanent magnets. He suggests the use of the bronze containing 20 per cent. of aluminum as telephone and telegraph conductors, believing that the bi-metallic character of the alloy will be found to be a corrective of self-induction. The principle of producing alloys by applying aluminous vapor in its nascent state is found to work with a long range of metals besides iron, and makes an exceedingly fine aluminum silver alloy, possessing valuable properties.—*The Dental Record.*

